# MiR-200c-3p aggravates gastric cell carcinoma via KLF6

**DOI:** 10.1007/s13258-021-01160-6

**Published:** 2021-09-15

**Authors:** Ying Wang, Kaijuan Lu, Weibing Li, Zhigang Wang, Jing Ding, Zeyu Zhu, Zhipeng Li

**Affiliations:** 1 Jiangsu Cancer Hospital and Jiangsu Institute of Cancer Research and The Affiliated Cancer Hospital of Nanjing Medical University, Nanjing, 210009 Jiangsu Province People’s Republic of China; 2 Qidong Hospital of TCM, Nantong, Jiangsu Province, People’s Republic of China; 3 Huaian Hospital, Huaian, Jiangsu Province People’s Republic of China

**Keywords:** Gastric cell carcinoma, MicroRNA-200c-3p, Krüppel like factor 6, Proliferation, Metastasis

## Abstract

**Background:**

Gastric cell carcinoma (GCC) is a common and high-incidence malignant gastrointestinal cancer that seriously threatens human life and safety. Evidences suggest that microRNAs (miRNAs) exhibit an essential role in regulating the occurrence and development of GCC, while the effects and possible mechanisms remain to be further explored.

**Objective:**

This study was designed to explore whether miR-200c-3p exerted its functional role in the growth and metastasis of GCC, and investigate the possible mechanisms.

**Methods:**

The expression levels of miR-200c-3p in GCC tissues and cell lines were detected by qRT-PCR analysis. The functional role of miR-200c-3p in the viability, proliferation, migration and invasion of GCC cells were evaluated by CCK-8, EdU, wound healing and Transwell assays. In addition, the candidate targets of miR-200c-3p was predicted and confirmed by dual-luciferase reporter assay. Moreover, the relationship between miR-200c-3p and target (Krüppel like factor 6, KLF6) was assessed by qRT-PCR and western blot assays. Besides, the expression levels of KLF6 in GCC cells were determined by qRT-PCR and western blot assays. Furthermore, the role of KLF6 in the viability, proliferation, migration and invasion of GCC cells mediated with miR-200c-3p mimics was evaluated by CCK-8, EdU, wound healing and Transwell assays.

**Results:**

In the present study, a new tumor promoting function of miR-200c-3p was disclosed in GCC. We found that the expression of miR-200c-3p was obviously increased in clinic GCC tissues and cell lines. In addition, down-regulation of miR-200c-3p suppressed cell viability, proliferation, migration, and invasion in GCC cells. Moreover, KLF6 was verified as a direct target of miR-200c-3p by binding its 3’-UTR. Additionally, KLF6 was remarkably decreased and was negatively associated with the miR-200c-3p expression in GCC cell lines. Furthermore, over-expression of KLF6 retarded the effects of miR-200c-3p on the growth and metastasis of GCC cell lines.

**Conclusions:**

MiR-200c-3p potentially played a tumor-promoting role in the occurrence and development of GCC, which may be achieved by targeting KLF6.

**Graphic abstract:**

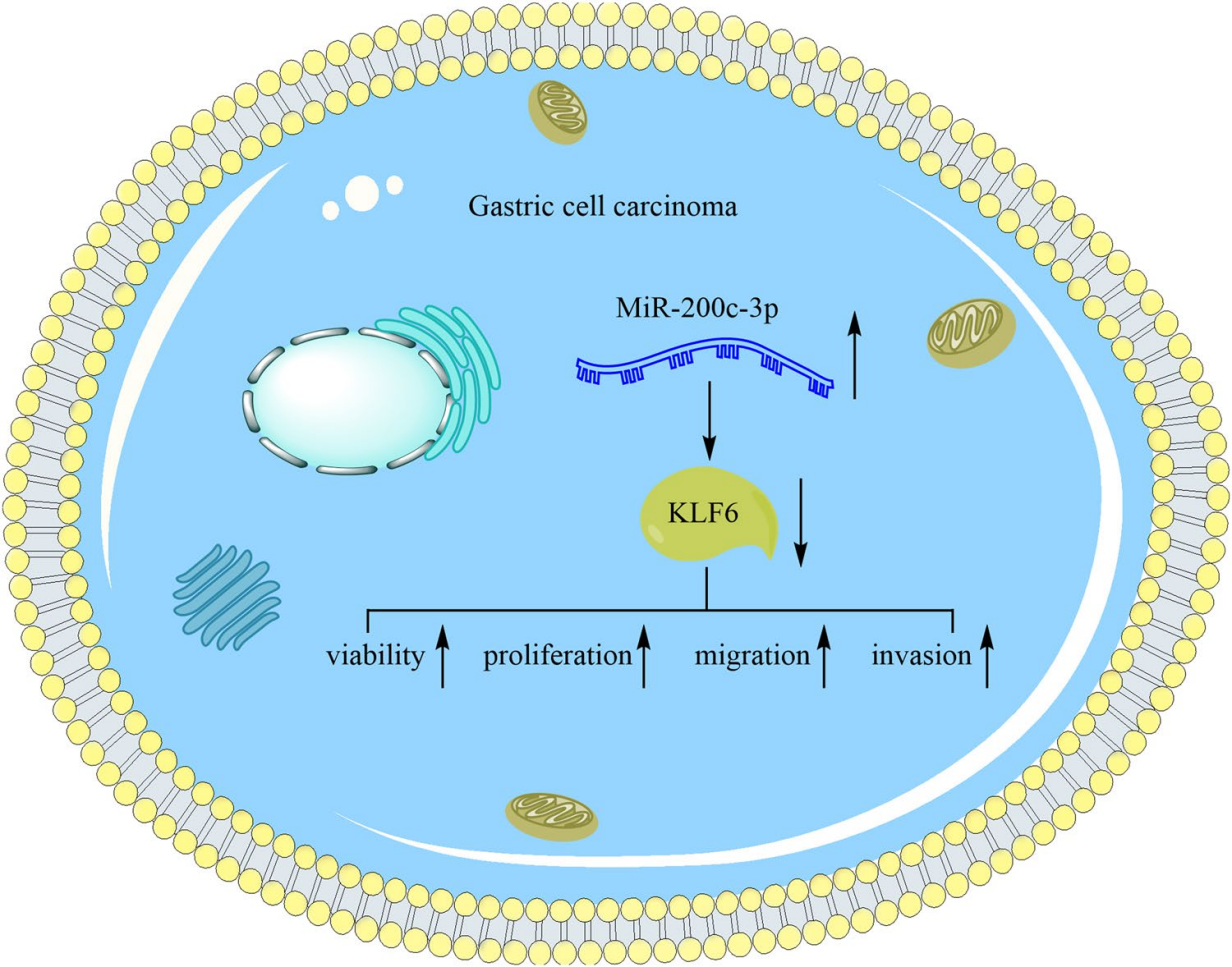

## Introduction

MicroRNAs (miRNAs) are highly conserved, and non-coding single stranded RNAs with about 19–22 nucleotides (Cui et al. 2017). MiRNAs were first found in *Caenorhabditiselegans*, and widely discovered in animals and plants (Snieckute et al. 2019). MiRNAs can regulate the gene expression by binding to the 3’-UTR of the target genes. If the nucleotide sequence of miRNAs is completely matched with the 3’-UTR regions of the target genes, the target gene can be degraded. If not, miRNAs can down-regulate the expression of target genes. Different miRNAs can co-regulate the same target gene, and different mRNAs can also be regulated and expressed by the same miRNA (Agarwal et al. 2015). As posttranscriptional gene regulatory factors, miRNAs are involved in a variety of cell physiological processes, including tumor genesis, differentiation, proliferation, apoptosis and cell cycle, but the detailed mechanisms of biological functions are not clear. MiRNAs can regulate gene expression and play an important role in a large number of physiological and pathological processes (Tutar 2014; Rupaimoole and Slack 2017). Human malignant tumors, including gastric cell carcinoma (GCC), usually show a large number of abnormal expressed genes. MiRNAs can regulate the expression of many coding genes, including oncogenes and tumor suppressor genes (Chen 2005; Sannicandro et al. 2020). The existing evidences show that the occurrence and development of various tumors including lung cancer, breast cancer, esophageal cancer and bladder cancer are closely related to the abnormal expression of miRNAs, thus miRNAs have therapeutic and diagnostic significance (Sharma and Sharma 2015; Li et al. 2018; Zhang et al. 2020). Moreover, in vitro experiments reveal that different cancer cell lines have different responses to the same miRNA, indicating that the target molecules of the same miRNA may be different in various cancers, or the activity of the target molecules of the miRNA may be different (Zheng et al. 2018; Chen et al. 2019).

GCC is one of the most common malignant gastrointestinal tumors, seriously threatening human health. According to the statistical data of *CA Cancer J Clin* in 2016, there are about 679,100 new cases of GCC and 498,000 deaths every year. The incidence rate and mortality rate of GCC in Chinese population rank second, and second only to lung cancer (Chen et al. 2016). The prognosis of patients with GCC is closely related to the tumor stage at the time of diagnosis. At current stage, about 50% of patients have cancer cell metastasis when they are diagnosed, therefore, the effect of treatment is relatively poor. Although great progress has been made in the diagnosis and treatment of GCC, it can only improve the treatment effect of GCC patients who are early detected, but not play a significant role in the treatment of GCC patients in middle and advanced stages (Liu and Xiao 2014; Matuszcak et al. 2014; Jiang et al. 2015; Wan et al. 2015). And the current treatment cannot significantly prolong the survival of GCC patients. Therefore, it is very important to study the mechanisms of GCC to screen the high-risk population and early patients from the molecular level, which may explore the cause of GCC and develop new treatment methods.

More and more studies show that the abnormal expression of miRNAs in GCC is closely related to the occurrence and development of tumors (Shin and Chu 2014). For example, up-regulating the expression of miR-17-5p/20a in GCC promotes the cell cycle process and inhibit the apoptosis of tumor cells (Wang et al. 2013). Besides, miR-106b and miR-93 have positive effects on attenuating TGF-β-induced apoptosis of GCC cells through inhibiting the expression of Bcl2L11 (Zhang et al. 2017). In addition, miR-150 promotes the growth of GCC cells by targeting EGR2 (Wu et al. 2010). Moreover, miR-204 is down-regulated in GCC cells, and its ectopic expression can inhibit the metastasis and invasion of GCC cells (Yuan et al. 2019). MiR-200c-3p, as a member of the miR-200 family, has been reported to be involved in the occurrence and progression of various cancers (Liu et al. 2018b). For instance, miR-200c-3p is declined in renal cell carcinoma tissues and cell lines. Overexpression of miR-200c-3p obviously suppresses proliferation, migration/invasion, and induced apoptosis of renal cell carcinoma cells through targeting SOX2 via modulating the wnt/β-catenin signaling pathway (Maolakuerban et al. 2018). In addition, the expression of miR-200c-3p is significantly down-regulated in nephroblastoma tissues and cells, and up-regulation of miR-200c-3p inhibits proliferation, migration and invasion of nephroblastoma cells by targeting FRS2 (Li et al. 2019b). Moreover, miR-200c-3p is down-regulated in prostate cancer tissues, and is negatively associated with pathologic T and N stage in prostate cancer. Overexpression of miR-200c-3p significantly suppresses the formation of migration and invasion in prostate cancer cells via targeting ZEB2 (Zhang et al. 2019). However, the effects and mechanisms of miR-200c-3p on GCC are not clear. Therefore, this study was designed to explore whether miR-200c-3p exerted its functional role in the growth and metastasis of GCC, and investigate the possible mechanisms.

## Materials and methods

### Collection of GCC tissues

Human GCC tissues were collected from 30 patients, and paracancerous tissues were isolated at the same time before any therapeutic intervention in the Jiangsu Cancer Hospital according to a standard protocol. All the patients were given the written informed consent and the study was approved by Ethics Committee of Nanjing Medical University. Fresh tissues were stored at 80 °C for further experiments.

### Cell lines and cell culture

The GCC cell BGC-823 and gastric mucosal epithelial cell GES-1 were obtained from American Type Tissue Culture Collection (Manassas, VA), and routinely cultured in Dulbecco’s modified Eagle’s medium (DMEM, Invitrogen, Carlsbad, CA) supplemented with 10% fetal bovine serum (FBS, Invitrogen, GrandIsland, NY) and 1% penicillin–streptomycin (Sigma-Aldrich, St. Louis, MO) in a humidified condition with 5% CO_2_ at 37 °C.

### Cell transfection

MiR-200c-3p inhibitor/inhibitor NC, miR-200c-3p mimic/mimic NC, empty vector, and pcDNA3.1 cloned with KLF6 were synthesized in Gene Pharma Company (Shanghai, China). Lipofectamine 2000 (Invitrogen) was performed for cell transfection based on the specification. After 24 h, relative expression levels of miR-200c-3p and KLF6 were determined by qRT-PCR assay.

### CCK-8 assay

Viability of GCC cells after transfection was assessed using CCK-8assay. BGC-823 cells at a density of 1 × 10^4^ were cultured in 96-well plates. After incubation for 0, 24, 48 and 72 h, respectively, cells were incubated with 10 μL of CCK-8 reagents for another 4 h at 37 °C. Then, the absorbance was detected at the wavelength of 490 nm (Multiskan MK3, ThermoS cientific, USA).

### EdU assay

The proliferation of GCC cells after transfection was assessed by EdU assay. BGC-823 cells (5 × 10^4^/well) were cultured in 24-well plates and transfected for 48 h. Then BGC-823 cells were fixed with 4% paraformaldehyde, Triton X-100 was used to permeabilize the nuclear membrane, and BGC-823 cells were blocked with goat serum for 1 h. Further, BGC-823 cells were stained according to the manufacturer’s suggestions.

### Wound healing assay

Cell migration of GCC cells after transfection was evaluated by wound healing assay. BGC-823 cells at a density with 1 × 10^5^/mL) were seeded in a 6-well plate and carefully wounded using a yellow pipette tip. Then cellular debris was removed by washing with DMEM. The crosses of each well were photographed under an OlympusIX-71 microscope (Japan) at 0 and 48 h, respectively.

### Transwell migration and invasion assays

Transwell chambers (Corning, NewYork, USA) were used to evaluate the migration and invasion of GCC cells after transfection. Briefly, BGC-823 cells (1 × 10^5^ per well) were added to the upper chamber of the inserts without or with matrigel, and 600 μL of medium with 20% FBS was added to the lower chamber. The migrated and invaded cells were stained by crystal violet and imaged after30 min of culturing.

### RNA extraction and qRT-PCR analysis

Total RNA was extracted from human GCC and GCC cells according to the manufacturer’s protocol. Then total RNA was reverse transcribed into cDNA using RNeasy plus microkit, which was used as starting materials for qRT-PCR on the Step One System (Life Technologies Corp). Primer sequences used were designed as follows: miR-200c-3p forward, 5′-CACTGGATTGGAGGAGGG-3′, and miR-200c-3p reverse, 5′-GAGCTTGACCACCGACTC-3′. KLF6 forward, 5′-AGTTAACCAGGCAC TTCCG-3′, KLF6 reverse, 5′-CTTTTAGCCTACAGGATCCACC-3′. U6 forward, 5′-CTCGCTTCG GCAGCACA-3′, U6 reverse, 5′-AACGCTTCACGAATTTGCGT-3′. β-actin forward, 5′-AGAAGGC TGGGGCTCATTTG-3′, β-actin reverse, 5′-AGGGGCCATCCACAGTCTTC-3′. Conditions for qRT-PCR were used: 95 °C for 10 min, 40 cycles of 95 °C for 15 s, and 60 °C for 1 min. All target gene transcripts were normalized to U6 or β-actin using the 2^−ΔΔCT^ method.

### Western blot analysis

The protein of GCC cells was lysed in RIPA lysis buffer. After 12,000*g* centrifugation for 15 min at 4 °C, the total protein concentrations were determined by BCA protein assay kit (Beyotime, Haimen, China). Equivalent samples were separated using 10% SDS-PAGE and then transferred onto a PVDF membrane for 2 h, and then blocked with 5% non fat skim milk in Tris-Buffered Saline and Tween-20 (TBS-T) buffer at room temperature for 1 h and incubated with the following primary antibodies at 4 °C overnight: KLF6 (ab241385, 1: 1, 000) and β-actin (ab8226, 1: 1, 000). After washing 3 times with TBS-T, the second antibody was added and incubated for 2 h at room temperature. Enhanced Chemiluminescence Detection System was carried out to evaluate the protein expressions. β-actin was used as the loading controls. Antibodies were purchased from Abcam (Cambridge, MA, USA).

### Target prediction and dual-luciferase reporter assay

The candidate target gene of miR-200c-3p was predicted with ENCORI, miRwalk and TargetScan, and KLF6 was chosen as a target gene. The wild type and mutant KLF6 3’-UTR dual-luciferase reporter vectors were constructed. Cells were transfected with 80 ng luciferase reporter vectors and miR-200c-3p mimic using the lipofectamine 2000 (Invitrogen, CA, USA). After 24 h, luciferase activities were measured using dual-luciferase reporter system (Berthold) according to the manufacturer’s instructions.

### Data analysis

All experiments were carried out at least three times, and the data were presented as the mean ± standard deviation (SD) and analyzed by GraphPad Prism 5.0 (LaJolla, CA, USA). The student’s *t* test or one-way ANOVA was used to measure statistical significance of differences between two groups or multiple groups, respectively. A *P* value < 0.05 was considered statistically significant.

## Results

### MiR-200c-3p is up-regulated in clinical GCC tissues and cell lines

**Fig. 1:**
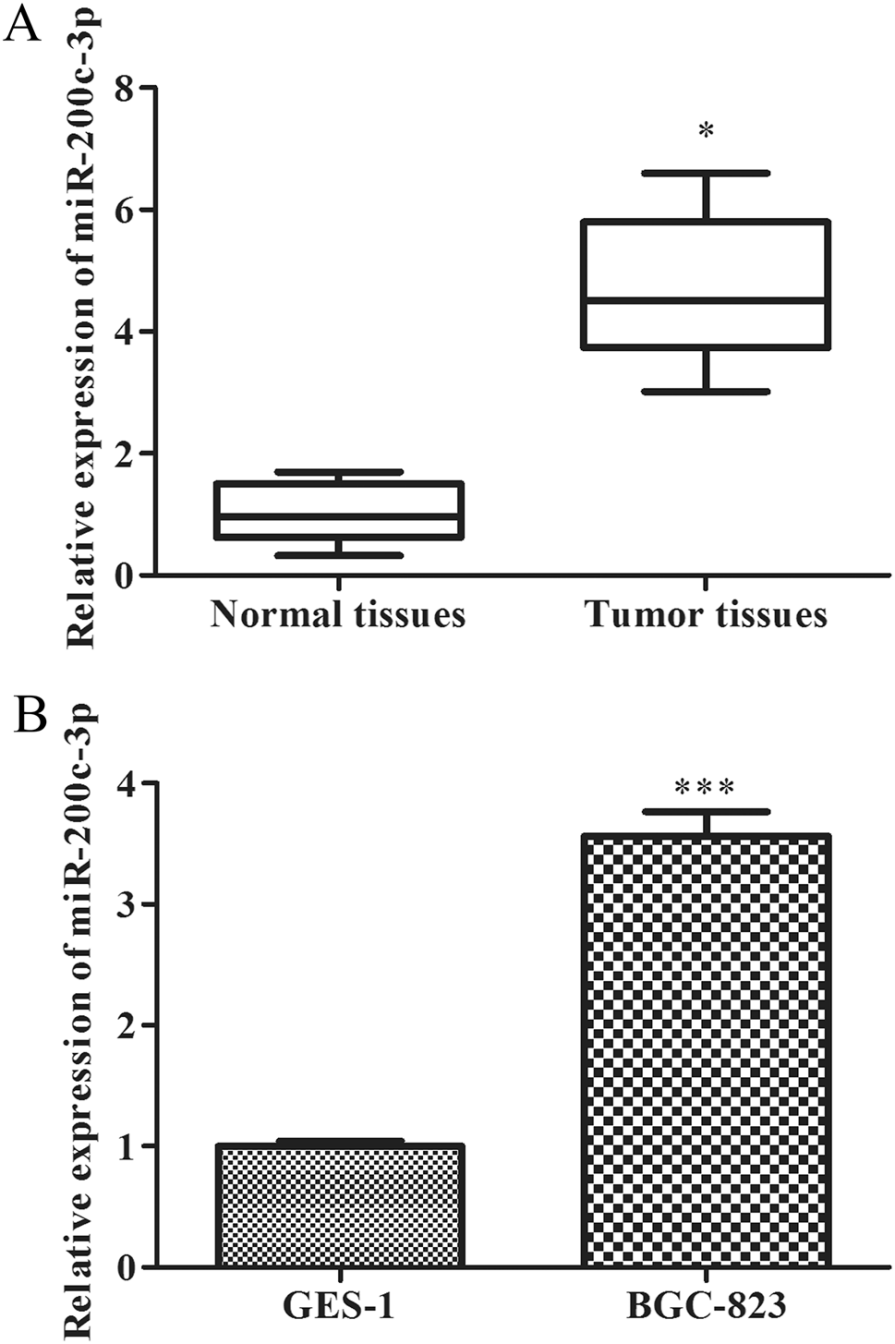
MiR-200c-3p is up-regulated in clinical GCC tissues and cell ines. **A** The expression of miR-200c-3p in GCC tissues (n = 30) was detected by qRT-PCR assay. *P < 0.05 *vs.* normal tissues. **B** The expression of miR-200c-3p in GCC cell lines was detected by qRT-PCR assay. ***P < 0.001 *vs.* GES-1 cells. All data were presented as mean ± SD

To investigate the effects and possible mechanisms of miR-200c-3p on the occurrence and progression of GCC, qRT-PCR was adapted to evaluate the expression of miR-200c-3p in GCC tissues (*n* = 30). As shown in Fig. 1A, the miR-200c-3p was overexpressed in clinical GCC tissues compared with that in paracancerous tissues. Furthermore, miR-200c-3p expression in GCC cells including BGC-823 was also assessed with qRT-PCR assay. As expected, miR-200c-3p expression in BGC-823 cells was significantly up-regulated relative to that in gastric mucosal epithelial cell GES-1 shown in Fig. 1B. These data suggested that miR-200c-3p might act as an oncogene in occurrence and progression of GCC.

### Down-regulation of miR-200c-3p inhibits viability, proliferation, migration and invasion in GCC cells

**Fig. 2:**
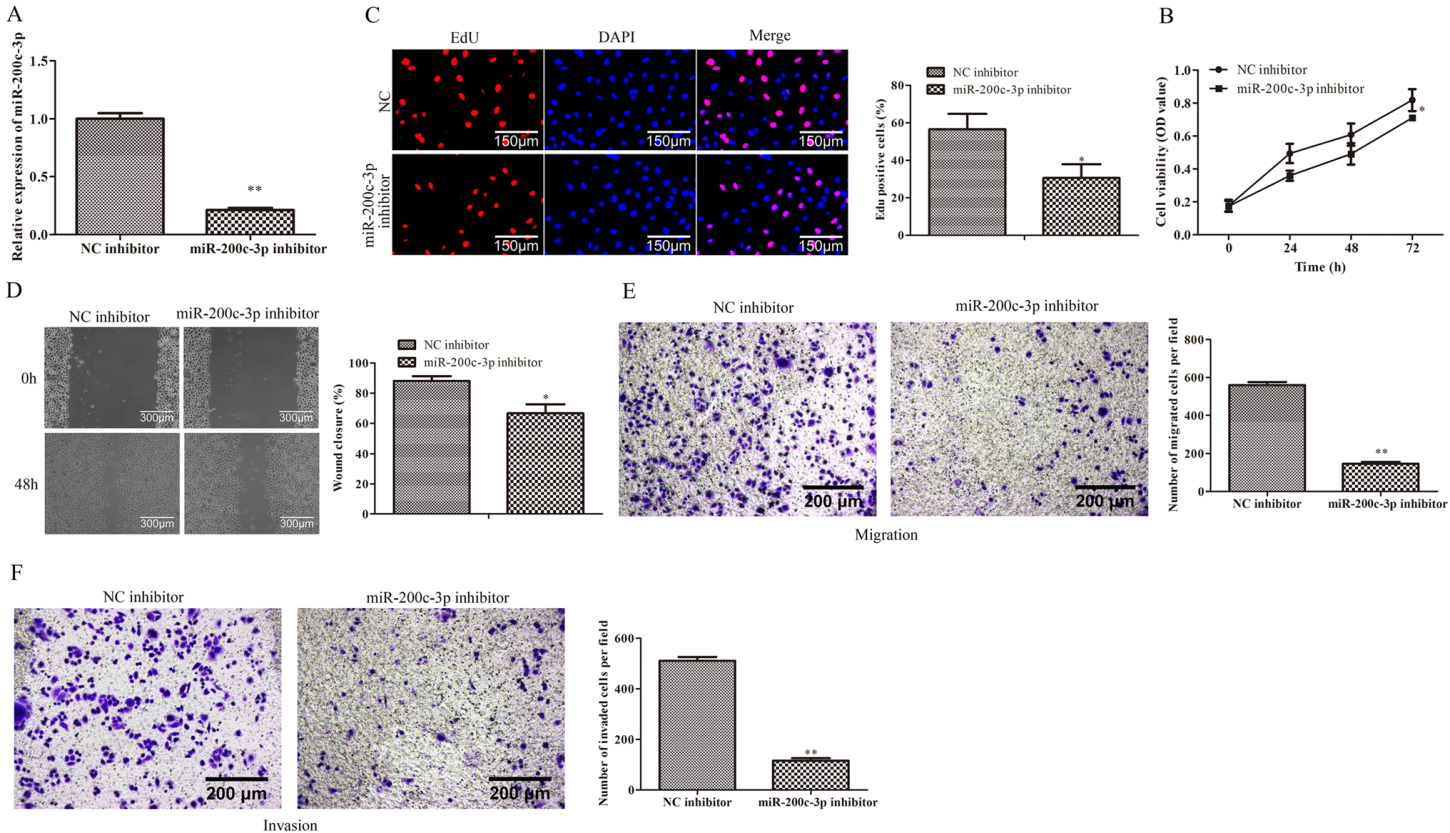
Down-regulation of miR-200c-3p inhibits viability, proliferation, migration and invasion in GCC cells. **A** qRT-PCR assays were performed to evaluate the miR-200c-3p expression in BGC-823 cells after transfection with miR-200c-3p inhibitor. **B** The viability of BGC-823 cells transfected with miR-200c-3p inhibitor was assessed by CCK-8 assay at indicated times. **C** The proliferation of BGC-823 cells transfected with miR-200c-3p inhibitor was evaluated by EdU assay. **D** The migration of BGC-823 cells transfected with miR-200c-3p inhibitor was determined by wound healing assay. **E** The migration of BGC-823 cells transfected with miR-200c-3p inhibitor was determined by Transwell migration assay. **F** The invasion of BGC-823 cells transfected with miR-200c-3p inhibitor was determined by Transwell invasion assay. *P < 0.05, **P < 0.01 vs. NC inhibitor. All data were presented as mean ± SD. *n* = 3

To further explore the effects of miR-200c-3p on GCC, BGC-823 cells were transfected with miR-200c-3p inhibitor and negative control, and qRT-PCR was performed. As indicated in Fig. 2A, the expression level of miR-200c-3p was notably reduced in BGC-823 cells transfected with miR-200c-3p inhibitor compared with NC inhibitor group. Moreover, CCK-8 assay was carried out to assess the effects of miR-200c-3p on the viability of BGC-823 cells. The data of Fig. 2B revealed that miR-200c-3p inhibitor significantly suppressed the viability of BGC-823 cells in a time-dependent manner, when compared with NC inhibitor group. In addition, the role of miR-200c-3p in the proliferation of BGC-823 cells was determined by EdU assay. The data of Fig. 2C showed that the EdU-positive cells in BGC-823 cells transfected with miR-200c-3p inhibitor were obviously reduced compared to NC inhibitor group. Moreover, the effects of miR-200c-3p on cell migration and invasion were evaluated by wound healing, and Transwell migration and invasion assays. As expected, the results of wound healing and Transwell migration assays (Fig. 2D and E) showed that down-regulation of miR-200c-3p remarkably inhibited the migration of BGC-823 cells relative to NC inhibitor group. Furthermore, the data of Transwell invasion assay illustrated that the number of invasion cells was obviously reduced after transfection with miR-200c-3p inhibitor in BGC-823 cells compared with NC inhibitor group (Fig. 2F). These data suggested that down-regulation of miR-200c-3p inhibits viability, proliferation, migration and invasion of GCC cells.

### KLF6 is direct target of miR-200c-3p in GCC cells

**Fig. 3:**
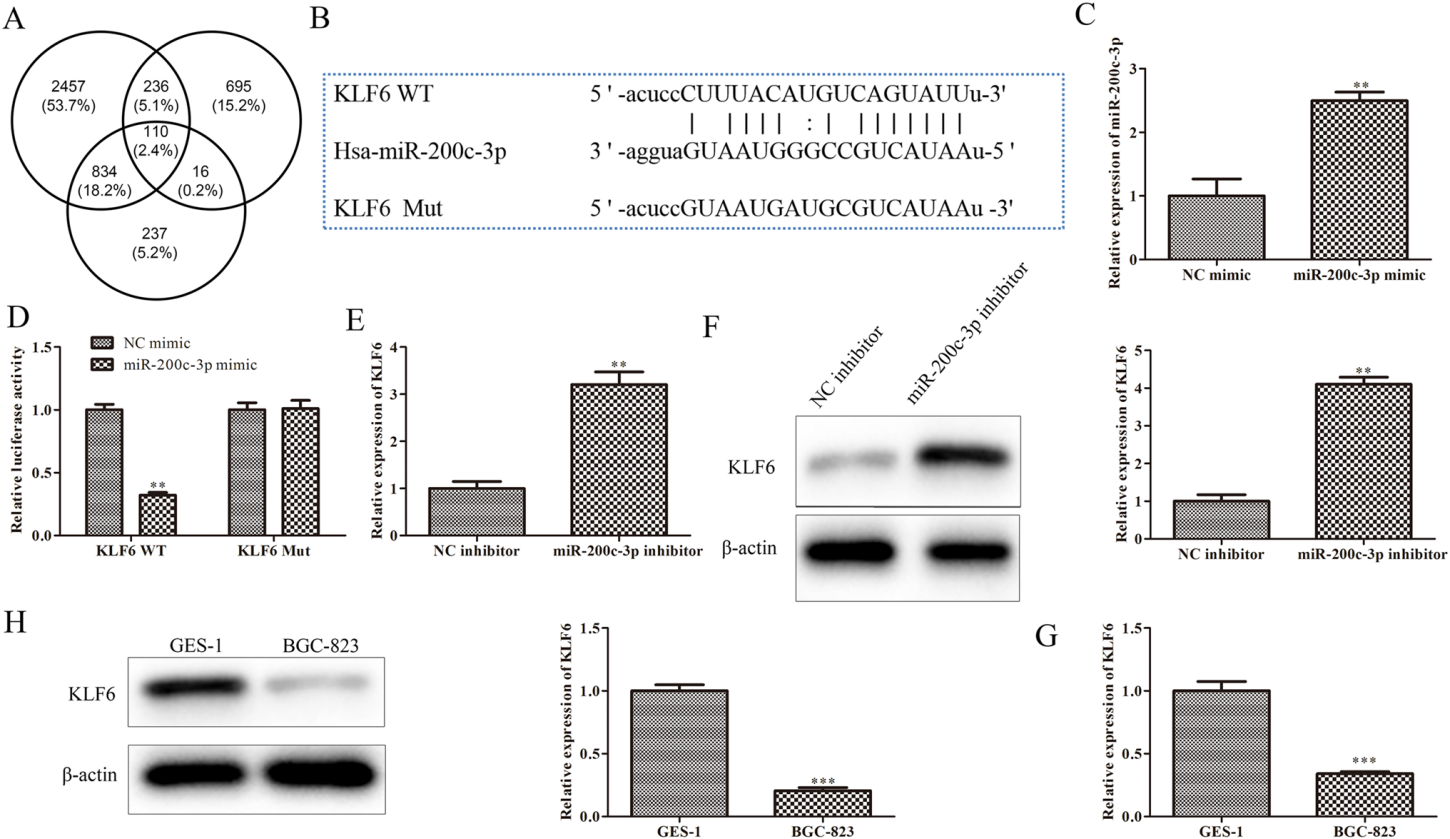
KLF6 is a direct target of miR-200c-3p in GCC cells. **A** The target gene of miR-200c-3p was predicted with ENCORI, miRwalk and Targetscan. **B** Binding sites between miR-200c-3p and KLF6. **C** qRT-PCR assay was performed to evaluate the miR-200c-3p expression in BGC-823 cells transfected with miR-200c-3p mimic. **P < 0.01 vs. NC mimic. **D** Dual luciferase reporter analysis was employed to validate the interactions between miR-200c-3p and KLF6. **P < 0.01 vs*.* NC mimic. **E** qRT-PCR assay was performed to evaluate the KLF6 expression in BGC-823 cells transfected with miR-200c-3p inhibitor. ^**^P < 0.01 vs. NC inhibitor. **F** Western blot assay was performed to evaluate the KLF6 expression in BGC-823 cells transfected with miR-200c-3p inhibitor. **P < 0.01 vs. NC inhibitor. **G** The expression of KLF6 in BGC-823 cells was detected by qRT-PCR assay. ***P < 0.001 vs. GES-1 cells. **H** The expression of KLF6 in BGC-823 cells was detected by western blot assay. ***P < 0.001 vs. GES-1 cells. All data were presented as mean ± SD. *n* = 3

In order to study on the possible target genes of miR-200c-3p involved in the occurrence and progression of GCC. Firstly, ENCORI, miRwalk and Targetscan were jointly performed, and a total of 110 intersecting target genes were selected (Fig. 3A). Among them, KLF6, a target gene related to GCC, was chosen for further study (Fig. 3B), since KLF6 functions as an important regulator controlling the occurrence and development of various tumors, including thyroid cancer, ovarian cancer and gastric cancer (Yang et al. 2019; Han et al. 2020; Li et al. 2020). To further confirm the targeting relationship between miR-200c-3p and KLF6, qRT-PCR was performed to detect transfection efficiency in BGC-823 cells. The data of Fig. 3C showed that the expression level of miR-200c-3p was significantly up-regulated in BGC-823 cells transfected with miR-200c-3p mimic. Then, dual-luciferase reporterassay was adapted and we found that exogenous expression of miR-200c-3p could distinctly weaken the luciferase activity of 3’-UTR of KLF6, whereas the inhibitory effect was blocked by mutation on the putative binding sites existed on the 3’-UTR of KLF6 (Fig. 3D). Furthermore, qRT-PCR and western blot assays were used to evaluate the mRNA and protein expression of KLF6 in BGC-823 cells transfected with miR-200c-3p inhibitor. As presented in Fig. 3E and F, the mRNA and protein expression levels of KLF6 were significantly increased in BGC-823 cells were transfected with miR-200c-3p inhibitor. Additionally, KLF6 mRNA and protein levels in BGC-823 cells were also assessed with qRT-PCR and western blot assays. As expected, KLF6 mRNA and protein levels in BGC-823 cells were significantly down-regulated relative to those in GES-1 cells shown in Fig. 3G and H. These data suggested that KLF6 may be a potential target gene of miR-200c-3p in GCC.

### KLF6 mediates the effects of miR-200c-3pon the GCC cells

**Fig. 4:**
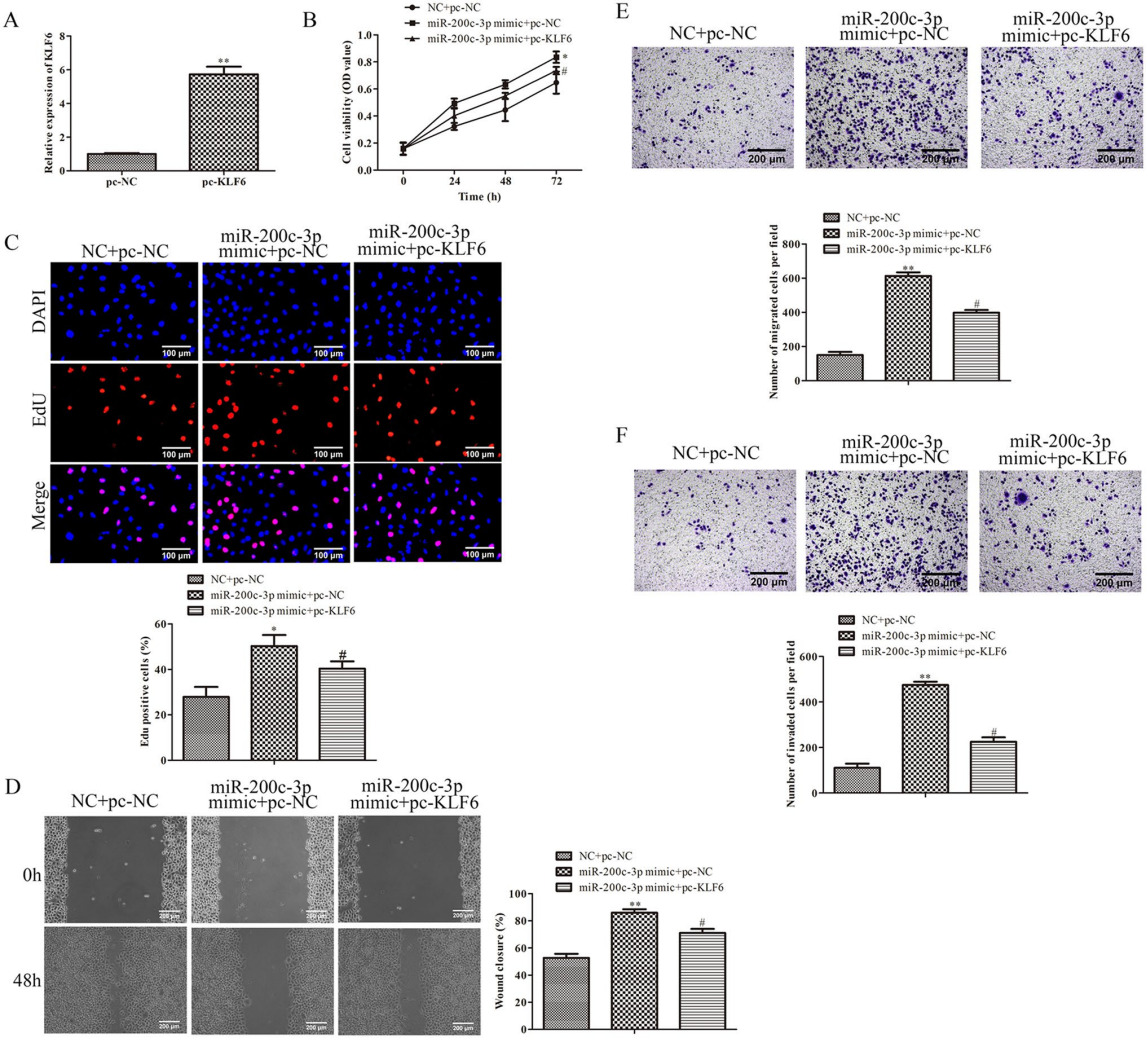
KLF6 mediates the effects of miR-200c-3pon the GCC cells. **A** The expression of KLF6 in BGC-823 cells transfected with pc-KLF6 was evaluated by qRT-PCR assay. **P < 0.01 vs. pc-NC. **B** The viability of BGC-823 cells transfected with pc-KLF6 was assessed by CCK-8 assay at indicated times. **C** The proliferation of BGC-823 cells transfected with pc-KLF6 was evaluated by EdU assay. **D** The migration of BGC-823 cells transfected with pc-KLF6 was determined by wounding healing assay. **E** The migration of BGC-823 cells transfected with pc-KLF6 was determined by Transwell migration assay. **F** The invasion of BGC-823 cells transfected with pc-KLF6 was determined by Transwell invasion assay.*P < 0.05, **P < 0.01, ***P < 0.001 vs*.* NC + pc-NC. ^#^*P* < 0.05, ^##^*P* < 0.01, ^###^*P* < 0.001 *vs.*miR-200c-3p mimic + pc-NC. All data were presented as mean ± SD. *n* = 3

To further assess whether miR-200c-3p functioned as an oncogene mediated with KLF6, BGC-823 cells were transfected with pc-KLF6, and the transfection efficacy was determined by qRT-PCR assay. As expected, the expression of KLF6 was obviously up-regulated in BGC-823 cells transfected with pc-KLF6 (Fig. 4A). Functionally, the result of CCK-8 assay illustrated that overexpression of KLF6 could obviously eliminate the prominent reduction in cell viability of BGC-823 cells transfected with miR-200c-3p mimic (Fig. 4B). EdU assay was also clarified that up-regulation of KLF6 could restore the increasing cell proliferation of BGC-823 cells transfected with miR-200c-3p mimic as shown in Fig. 4C. Correspondingly, wound healing and Transwell assays manifested that over-expression of KLF6 could partly restore the promoting effects of miR-200c-3p mimic on the migration and invasion of BGC-823 cells (Fig. 4D–F). These data suggested that KLF6 is an important mediator linking the function of miR-200c-3pto the progression of GCC.

## Discussion

In recent years, researchers have focused on the relationship between abnormal expression of miRNAs and cancer (Tutar et al. 2018). MiRNAs participate in many physiological and pathological processes through completely or incompletely complementary between seed region and 3′-UTR of target genes (Liu et al. 2018a). Many studies have shown that many kinds of miRNAs are abnormally expressed in tumor tissues, and the expression level is closely related to the occurrence and development of tumors (Behl et al. 2020). In the study, we found that miR-200c-3p was closely up-regulated in clinical GCC tissues and cell lines. Studies have demonstrated that miR-200c-3p was high-expressed in many tumors, including colorectal cancer, breast cancer, prostate cancer, which was in consistent with our results, suggesting that miR-200c-3p might act as an oncogene in occurrence and progression of GCC.

In order to study the effects of miR-200c-3p on the viability, proliferation, migration and invasion of GCC cells, CCK-8, EdU, wound healing, Transwell migration and invasion assays were carried out. As expected, down-regulation of miR-200c-3p could inhibit viability, proliferation, migration and invasion of GCC cells. There was moderate evidence suggesting positive effects of miR-200c-3p on the growth and metastasis of other tumors. For example, miR-200c-3p showed inhibitory effects of migration and invasion of renal cell carcinoma cells through targeting SOX2 (Li et al. 2019a). In addition, miR-200c-3p inhibited proliferation, migration, and invasion of nephroblastoma cells via targeting FRS2 (Li et al. 2019b). Moreover, miR-200c-3p was found to remarkably inhibit the formation of migration and invasion in prostate cancer cells via suppression of E-cadherin-induced Epithelial-mesenchymal transition (EMT), achieved by targeting ZEB2 (Zhang et al. 2019). These findings are basically consistent with those in this study, suggesting that the abnormal expression of miR-200c-3p in GCC may play an important role in the progression of GCC.

This study predicted that the 3′-UTR region of Krüppel like factor 6 (KLF6) contained complementary sites to miR-200c-3p, suggesting that KLF6 might be a target gene of miR-200c-3p. Zinc finger (ZNF) structure is the most representative eukaryotic transcription factor in a large number of DNA binding motifs, and Cys2His2 ZNF gene sequence is the most standardized type (Jen and Wang 2016). KLF family is a kind of ZNF structure, which is also important for tumorigenesis (Lu et al. 2015). KLF6 a nuclear transcription regulatory factor commonly expressed in mammals, belonging to the KLF family, which is located in human chromosome 10q15 and has a total length of about 7000 bases (Dumayne et al. 2020). In recent years, it has been found that KLF6 is a nuclear transcription regulator involved in the regulation of growth and development, cell proliferation, differentiation and angiogenesis (Shi et al. 2020). KLF6 is initially considered as a tumor suppressor in prostate cancer, and is regulated through activating CDHL promoter (Tian et al. 2020). Additionally, the above tumor suppressive function of KLF6 is further confirmed in hepatocellular carcinoma and gastric cancer, and is related to the effect of cell differentiation (He et al. 2017; Luo et al. 2019; Tian et al. 2019). In this study, KLF6 was reduced in GCC cells, and was a target of miR-200c-3p confirmed by dual-luciferase reporter assay, which were consistent with the bioinformatic prediction. Moreover, the mechanisms of miR-200c-3p in regulating KLF6 were further explored through rescue experiment. As expected, overexpression of KLF6 could partly restore the effects of miR-200c-3p on viability, proliferation, migration and invasion of GCC cells.

In conclusion, the expression of miR-200c-3p in clinical GCC tissues and cell lines was significantly higher, and miR-200c-3p negatively regulated the expression of KLF6 by acting on the 3’-UTR region of KLF6, thus inhibiting the growth and metastasis of GCC cells, which will provide research basis for gene targeted therapy of GCC in the future.
